# Bioinformatics Analysis of Hypoxia‐Related Mechanisms in Endometriosis: DDR2 as a Potential Diagnostic and Therapeutic Biomarker

**DOI:** 10.1155/bmri/4765791

**Published:** 2025-12-18

**Authors:** Mingqi Zhao, Panpan Zhao, Caiyi Wang, Dan Ren, Yuxia Song, Xiaoqin Lu

**Affiliations:** ^1^ Reproductive Hospital of the Second Affiliated Hospital of Zhengzhou University, Zhengzhou, Henan, China; ^2^ Department of Obstetrics and Gynecology, The Second Affiliated Hospital of Zhengzhou University, Zhengzhou, Henan, China, zzusah.com

**Keywords:** DDR2, endometriosis, hypoxia, machine learning

## Abstract

**Background:**

Endometriosis is a chronic gynecological disorder characterized by the presence of endometrial‐like tissue outside the uterine cavity, causing chronic pain and infertility. Hypoxia plays a significant role in the progression of endometriosis.

**Methods:**

We performed bioinformatics analysis on GEO datasets to identify differentially expressed genes (DEGs) in endometriosis, using weighted gene coexpression network analysis (WGCNA)and GeneCards for hypoxia‐related genes. Machine learning models identified key hub genes. CCK‐8, EdU, and Transwell assays assessed cell proliferation, migration, and invasion. Molecular docking was performed to investigate the interactions between the drug and the protein.

**Results:**

In the GEO dataset analysis, 2834 DEGs were identified. Using WGCNA, a green module strongly correlated with endometriosis was identified. Intersecting this module with the hypoxia‐related genes resulted in the selection of 449 key genes. Machine learning models, including support vector machines (SVMs), were employed to identify hypoxia‐related DEGs with significant predictive value. LASSO and SVM‐RFE were used to refine this list, ultimately selecting six hub genes: DDR2, ENO3, ESM1, NMBR, PRKAB1, and PRPF19. Validation with an independent dataset confirmed DDR2 as a promising diagnostic biomarker. Functional assays demonstrated that DDR2 knockdown significantly inhibited cell proliferation, migration, and invasion in the endometriosis cell lines VK2/E6E7 and 12Z. DDR2, a receptor tyrosine kinase, mediates extracellular matrix remodeling and cell invasion under hypoxia. By interacting with collagen and HIFs, DDR2 activates pathways that promote MMP secretion, angiogenesis, and migration, facilitating endometriotic cell progression in the hypoxic microenvironment. Molecular docking identified key amino acids near DDR2′s binding pocket that form hydrophobic interactions, hydrogen bonds, and *π*‐stacking with baicalein, cavidine, sitogluside, and stigmasterol, further supporting DDR2′s potential as a therapeutic target.

**Conclusion:**

DDR2 is a key hypoxia‐related gene in endometriosis and a promising diagnostic and therapeutic biomarker.

## 1. Introduction

Endometriosis is a chronic gynecological disorder defined by the presence of endometrial‐like tissue outside the uterine cavity, accompanied by inflammation, angiogenesis, and fibrosis. Clinically, it manifests as chronic pelvic pain, dysmenorrhea, dyspareunia, and infertility, leading to substantial impairment in patients′ quality of life and well‐being [1]. Affecting approximately 10% of reproductive‐age women—equivalent to 190 million individuals worldwide—the true prevalence is likely underestimated due to diagnostic challenges, as definitive diagnosis often requires invasive laparoscopy [2]. While higher detection rates are observed in regions with advanced healthcare systems due to better diagnostic capabilities, underreporting remains prevalent in low‐resource settings. Ethnic and geographic disparities in detection further stem from genetic predisposition and unequal access to healthcare [3]. Symptoms typically begin during adolescence, yet diagnostic delays of 7–10 years are common due to the nonspecific nature of symptoms and cultural stigmas surrounding menstruation and pelvic pain. Emerging diagnostic tools, such as genomic classifiers, offer promise for early and accurate detection, but they remain largely experimental [4]. Beyond its physical burden, endometriosis imposes profound psychological and economic impacts, including reduced work productivity, increased risks of anxiety and depression, and diminished sexual health. The global economic burden, encompassing direct medical expenses and indirect productivity losses, is estimated to reach billions annually, emphasizing the urgent need for improved diagnostic strategies and therapeutic interventions [5].

The pathogenesis of endometriosis is a complex, multifactorial process involving interrelated genetic, hormonal, immune, and environmental mechanisms. The retrograde menstruation theory posits that menstrual endometrial cells implant ectopically, yet it fails to account for cases involving distant lesions, such as those in the lungs [6]. The metaplasia theory suggests that mesothelial cells undergo transformation into endometrial‐like tissue, while the Müllerian remnant theory attributes lesions to embryonic duct remnants persisting into adulthood [7, 8]. Immune dysfunction further exacerbates the condition by enabling misplaced endometrial cells to evade clearance, survive, and proliferate. Concurrently, hormonal dysregulation, including estrogen‐driven proliferation and progesterone resistance, plays a critical role in lesion growth and persistence [9, 10]. Genetic predisposition, evidenced by loci such as WNT4 and familial clustering, highlights heritable contributions to the disease [11]. Despite these valuable insights, the complex interactions between these factors are still not fully understood, hindering progress in diagnosis and treatment. Moreover, delays in diagnosis, caused by nonspecific symptoms, continue to pose significant challenges in managing the disease effectively [12].

The hypoxic microenvironment, characterized by oxygen levels below 1%–2%, plays a pivotal role in various pathophysiological conditions, including tumors, chronic inflammation, and ischemia. Under hypoxic conditions, metabolic reprogramming occurs, driven by hypoxia‐inducible factors (HIFs), particularly hypoxia‐inducible Factor 1‐alpha (HIF‐1*α*). HIF‐1*α* promotes a metabolic shift from oxidative phosphorylation to glycolysis, increasing lactate production and acidosis, while simultaneously suppressing mitochondrial respiration and altering lipid metabolism to adapt to oxygen deprivation [13, 14]. Beyond metabolism, HIFs regulate key genes involved in angiogenesis, tissue remodeling, and cell survival. Through the upregulation of vascular endothelial growth factor (VEGF), HIFs drive angiogenesis, ensuring oxygen and nutrient supply to hypoxic tissues. Additionally, HIFs enhance cell invasiveness and tissue remodeling by promoting epithelial–mesenchymal transition (EMT) and activating matrix metalloproteinases (MMPs), processes critical for metastasis and disease progression [15–17]. These combined adaptations enable cellular survival, migration, and pathological remodeling, establishing HIFs as central mediators of hypoxia‐driven diseases and potential therapeutic targets [18].

Hypoxia plays a pivotal role in the pathogenesis of gynecological diseases, influencing angiogenesis, inflammation, fibrosis, and tissue remodeling. In malignant conditions such as ovarian and endometrial cancers, HIF‐1*α* stabilizes VEGF, driving angiogenesis, EMT, and metastasis. Concurrently, hypoxia creates an acidic microenvironment that fosters aggressive tumor phenotypes, further promoting disease progression [19]. Beyond malignancies, hypoxia significantly impacts noncancerous gynecological conditions, including uterine fibroids and adenomyosis. In fibroids, HIF‐1*α* activation stimulates profibrotic pathways, leading to extracellular matrix (ECM) accumulation and fibroblast proliferation [19]. Similarly, in adenomyosis, hypoxia exacerbates inflammation and fibrosis through cytokines such as IL‐6 and TNF‐*α*, which activate TGF‐*β* signaling, driving a cycle of chronic inflammation, ECM remodeling, and lesion progression. Hypoxia‐regulated factors, including VEGF and miR‐210, serve as both biomarkers and therapeutic targets, underscoring the critical role of hypoxia in the progression of gynecological pathologies [19, 20].

The hypoxic microenvironment is a hallmark of ectopic endometriotic lesions and plays a crucial role in the pathophysiology and progression of endometriosis. Quantitative oxygen sensor studies have revealed significantly lower oxygen levels in ectopic lesions compared to eutopic endometrial tissues, providing direct evidence of hypoxia in these regions [21]. HIF‐1*α*, a key regulator of cellular adaptation to low oxygen levels, is highly stabilized in ectopic tissues, activating genes involved in angiogenesis, inflammation, and tissue remodeling. Hypoxia‐induced stress also increases the production of reactive oxygen species (ROS) and elevates the expression of hypoxia‐responsive microRNAs, such as miR‐210, which promote cell survival in adverse conditions [22, 23]. Mechanistically, hypoxia drives disease progression by upregulating proinflammatory cytokines like IL‐6 and TNF‐*α*, sustaining chronic inflammation through immune cell recruitment [24]. Simultaneously, HIF‐1*α* induces VEGF, stimulating the formation of new, yet frequently dysfunctional, blood vessels, which perpetuate hypoxia and create a cycle of inflammation and angiogenesis [22]. Additionally, hypoxia promotes EMT, enabling endometrial epithelial cells to acquire migratory and invasive properties through altered cadherin expression and activation of MMPs, facilitating the invasion of ectopic lesions into surrounding tissues [21].

This study is aimed at systematically investigating the role of hypoxia‐related genes in endometriosis by integrating bioinformatics analyses with advanced machine learning techniques, ultimately identifying key candidates for functional validation. Among these, discoidin domain receptor Tyrosine Kinase 2 (DDR2) emerged as a critical hypoxia‐related gene through a multistep pipeline combining weighted gene coexpression network analysis (WGCNA), differential expression analysis, and machine learning models. DDR2 was subsequently prioritized as a potential therapeutic target and subjected to experimental validation to confirm its functional significance in endometriosis.

## 2. Materials and Methods

### 2.1. Data Collection

The GSE7305, GSE11691, and GSE58178 datasets were retrieved from the Gene Expression Omnibus (GEO) database. Each dataset was selected based on the availability of paired normal and endometriosis samples, with consistent platform types to minimize technical variability. The GSE7305 dataset included 10 normal endometrial samples and 10 endometriosis samples, analyzed using the GPL570 [HG‐U133_Plus_2] Affymetrix Human Genome U133 Plus 2.0 Array. The GSE11691 dataset contained nine normal and nine endometriosis samples, analyzed on the GPL96 [HG‐U133A] Affymetrix Human Genome U133A Array. The GSE58178 dataset contained six normal and six endometriosis samples, analyzed using the GPL6947 Illumina HumanHT‐12 V3.0 Expression BeadChip. All datasets underwent normalization to ensure comparability across the studies. Hypoxia‐related gene sets were obtained from GeneCards (https://www.genecards.org/), with a relevance score greater than 1 as the inclusion criterion. This search resulted in the identification of 2786 genes associated with hypoxia for further analysis [25, 26].

### 2.2. Differentially Expressed Gene (DEG) Analysis

The analysis was performed using the R programming language and the limma package, which applies a linear model to calculate fold change (FC) values and adjusted *p* values using the Benjamini–Hochberg correction to control for false discovery rates (FDRs). Genes with |log2FC| > 1 and FDR‐adjusted *p* value < 0.05 were considered significantly differentially expressed. Heatmaps and volcano plots were generated to visualize the expression profiles of the identified DEGs. These DEGs served as the foundation for subsequent analyses, including functional enrichment, machine learning, and integrative bioinformatics.

Receiver operating characteristic (ROC) curve analysis was performed to evaluate the diagnostic performance of the identified key genes in distinguishing ectopic from eutopic endometrial tissues. ROC curves were generated using the pROC package in R, and the area under the curve (AUC) was calculated to assess the predictive accuracy of the model.

### 2.3. Gene Ontology (GO) and Kyoto Encyclopedia of Genes and Genomes (KEGG) Enrichment Analysis

GO and KEGG enrichment analyses were conducted to identify the biological functions and pathways associated with DEGs. Using the R package clusterProfiler, DEGs were analyzed for enrichment in biological processes (BPs), cellular components (CCs), molecular functions (MFs), and pathways.

### 2.4. WGCNA

WGCNA was conducted to identify gene modules significantly associated with endometriosis and hypoxia. The normalized gene expression matrix from GEO datasets was used as input, with low‐variance genes excluded to enhance network stability and reduce noise. Using the WGCNA R package, pairwise correlations between genes were computed and converted into an adjacency matrix. A soft‐thresholding power of 8 was selected based on scale‐free topology criteria to ensure the construction of a reliable and biologically meaningful network. The topological overlap matrix (TOM) was subsequently generated to evaluate gene connectivity and similarity within the network. Hierarchical clustering was applied to group genes into distinct coexpression modules, each assigned a unique color for visualization. Module eigengenes (MEs), defined as the first principal component of each module, were calculated to summarize module expression profiles.

### 2.5. Machine Learning Models

To construct predictive models based on the expression profiles of hub genes, four machine learning algorithms were utilized: eXtreme Gradient Boosting (XGB), generalized linear model (GLM), random forest (RF) model, and support vector machine (SVM) model. These models were implemented using the “caret” R package. XGB, a robust boosting algorithm, builds models iteratively by optimizing the objective function and correcting residual errors, making it highly effective for complex datasets. GLM, an extension of linear regression, allows for the analysis of nonnormally distributed response variables through appropriate link functions and ensures a parsimonious representation of relationships between variables. SVM operates by maximizing the decision boundary margin and solving a convex quadratic programming problem, making it particularly suitable for high‐dimensional datasets. RF constructs an ensemble of decision trees through random sampling of training data, combining their outputs to enhance model accuracy and robustness.

The dataset was split into a training set (70%) and a validation set (30%) for model construction and evaluation. The “DALEX” package was used to interpret and visualize the residual distributions and feature importance across all models. Model parameters were fine‐tuned using the “caret” package with automated optimization, and fivefold cross‐validation was applied to assess model performance.

### 2.6. Feature Selection Using Least Absolute Shrinkage and Selection Operator (LASSO) and Support Vector Machine–Recursive Feature Elimination (SVM‐RFE)

Machine learning methods, including LASSO and SVM‐RFE, were applied to identify key genes from the DEGs. LASSO regression was performed using the glmnet package in R to select the most informative genes by shrinking the regression coefficients and eliminating irrelevant features. Genes with nonzero coefficients were retained for further analysis. Subsequently, SVM‐RFE was employed to rank the selected genes based on their importance in classification. This was implemented using the e1071 package, which recursively eliminates the least significant features to optimize the model′s performance. Cross‐validation was performed to ensure the robustness of the feature selection process.

### 2.7. Immune Infiltration Analysis

Immune infiltration analysis was performed using the CIBERSORT algorithm to evaluate the proportions of immune cell types in endometriosis samples. Gene expression profiles from selected datasets were input into the CIBERSORT tool, which deconvolutes bulk RNA data to estimate the relative abundance of 22 immune cell types.

The relative proportions of immune cells, such as macrophages, T cells, and natural killer cells, were compared between ectopic and eutopic endometrial tissues. Visualizations, including boxplots and correlation heatmaps, were generated to display immune cell composition differences.

### 2.8. Molecular Docking

Small molecules were selected from the TCMSP database. Their structures were prepared in AutoDockTools‐1.5.7, adding hydrogen atoms, computing charges, and exporting as PDBQT files. Protein structures (PDB: 6FER) and others were downloaded from the RCSB PDB database and processed using PyMOL and AutoDockTools‐1.5.7, removing water molecules and adding hydrogens. AlphaFold‐predicted structures were processed similarly. Docking simulations were performed using AutoDock Vina, generating nine conformations per ligand. The binding strength was evaluated by the docking score (Affinity), where values < −7 kcal/mol indicated strong binding. The lowest binding free energy conformations were selected for further analysis. Noncovalent interactions were analyzed with PLIP and visualized using PyMOL. The best docking models with binding free energy < −6 kcal/mol were selected for further study.

### 2.9. Cell Lines and Culture Conditions

The endometriosis cell lines used in the study (VK2/E6E7 and 12Z) were purchased from Procell Life Technology Co. Ltd. (Wuhan, China). The cells were cultured in high‐glucose Dulbecco ^′^s modified Eagle ^′^s medium (DMEM, Procell, PM15021, China) with 10% fetal bovine serum (Gibco, 10099141C, United States). The cells were cultured in a constant temperature incubator at 37°C and 5% CO_2_.

### 2.10. CCK‐8 Assay

Cells were seeded in 96‐well plates at a density of 1 × 10^4^ cells per well and cultured overnight. The CCK‐8 reagent (Abbkine, United States) was then added to each well (10 *μ*L per well), and the cells were incubated for 1–4 h at 37°C. Absorbance was measured at 450 nm using a microplate reader. Cell viability was determined by comparing the absorbance values to a control group.

### 2.11. 5‐Ethynyl‐2 ^′^‐Deoxyuridine (EdU) Assay

Cells were cultured in 6‐well plates and treated as necessary. EdU was added to the culture medium at a final concentration of 10 *μ*M and incubated for 2 h at 37°C. After incubation, cells were fixed with 4% paraformaldehyde and permeabilized with 0.5% Triton X‐100. EdU incorporation was detected using a Click‐iT EdU Imaging Kit (ServiceBio, China) according to the manufacturer ^′^s instructions. The results were visualized under a fluorescence microscope.

### 2.12. Transwell Assay

Cell migration and invasion were assessed using Transwell chambers with an 8‐*μ*m pore size. For migration, cells (1 × 10^5^) were seeded in the upper chamber with serum‐free medium, and the lower chamber was filled with complete medium as a chemoattractant. After 24 h of incubation at 37°C, nonmigrated cells were removed, and the migrated cells on the membrane were fixed, stained with crystal violet, and counted under a microscope. For invasion, the procedure was similar, but the upper chamber was coated with Matrigel (BD Biosciences). Data were presented as the number of cells that migrated or invaded per field.

### 2.13. Statistical Analysis

All statistical analyses were performed using R software (Version 4.3.1). For comparisons between groups, Student′s *t*‐test was applied when normality assumptions were met; otherwise, the Wilcoxon rank‐sum test was used. Spearman′s correlation coefficient was employed to evaluate the relationship between gene expression and immune cell infiltration, with statistical significance defined as *p* < 0.05.

## 3. Results

### 3.1. GEO Dataset Analysis

The GEO datasets GSE7305, GSE11691, and GSE58178 were retrieved from the GEO database and integrated for downstream analyses. Following normalization, boxplots of the processed data confirmed that the medians across all samples were comparable, indicating uniformity in preprocessing (Figures 1a, 1b, 1c, and 1d). Differential gene expression was visualized using a volcano plot (Figure 1e), while a UMAP plot demonstrated distinct clustering between “normal” and “endometriosis” samples, highlighting transcriptional differences between the groups.

Figure 1Normalization and differential expression analysis of gene expression datasets. (a–d) Boxplots and UMAP plots of gene expression before and after normalization for four different datasets: GSE7305, GSE11691, GSE58178, and the merged dataset. UMAP plots demonstrate the clear separation between normal endometrial tissue (blue) and endometriosis tissue (red) after normalization. (e) Heatmap showing the Top 10 most upregulated and downregulated genes from the merged dataset. (f) Venn diagram showing the overlap between differentially expressed genes (DEGs) and hypoxia‐related genes. Statistical thresholds for differential gene expression screening: DEGs were identified using a significance threshold of |logFC| > 0.3 and *p* < 0.05.

**Figure:**
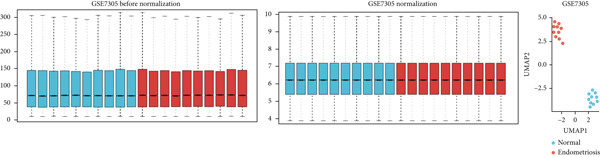
(a)

**Figure:**
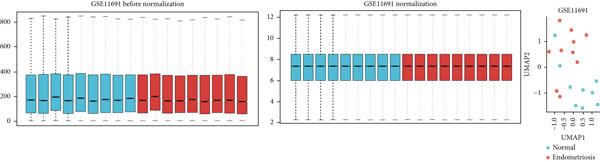
(b)

**Figure:**
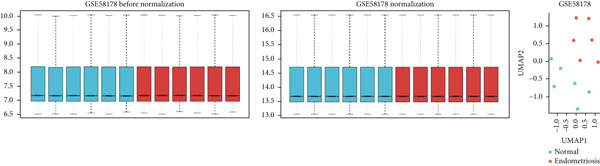
(c)

**Figure:**
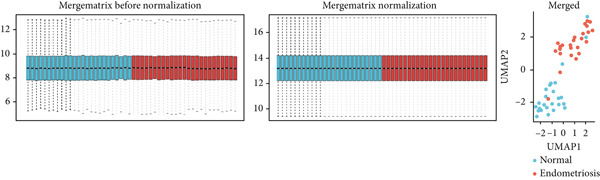
(d)

**Figure:**
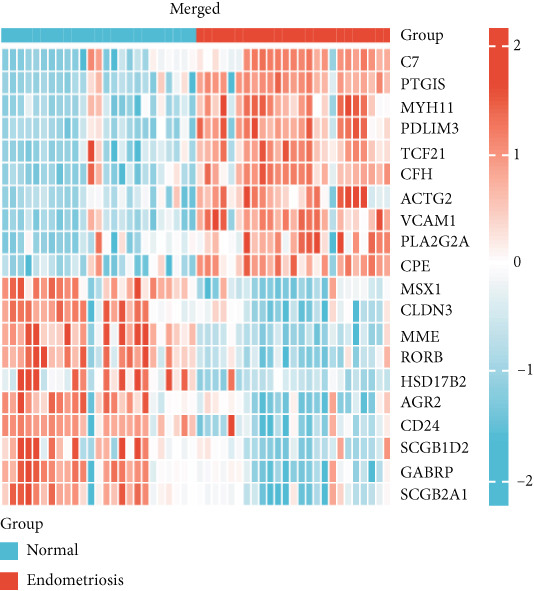
(e)

**Figure:**
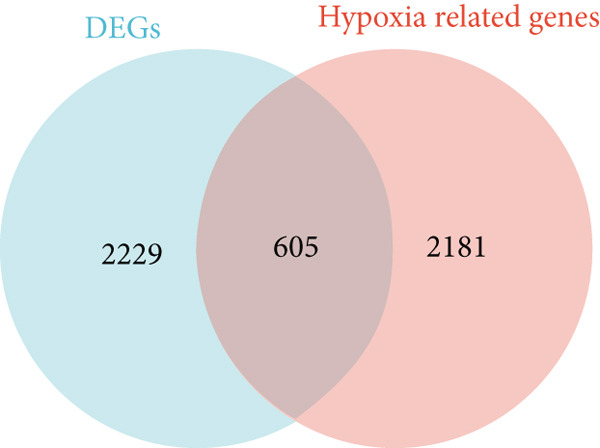
(f)

A heatmap was generated to display the Top 10 most upregulated and downregulated genes from the merged dataset (Figure 1e). From the combined dataset, 2834 DEGs were identified based on the criteria |logFC| > 0.3 and *p* < 0.05. Additionally, 2786 hypoxia‐related genes were obtained from the GeneCards database (relevance score > 1). By intersecting the DEGs with the hypoxia‐related genes, 605 overlapping genes were identified for further analysis (Figure 1f).

### 3.2. WGCNA

The expression profiles of all genes from 25 endometriosis samples and 25 normal endometrial samples were utilized to construct coexpression modules using the WGCNA algorithm. To ensure robust analysis, the top 25% of genes with the highest variance were selected for further investigation. Cluster analysis of the samples was performed with the “flashClust” package. When the soft‐thresholding power was set to 8, the scale‐free topology fit index reached 0.85, and the network′s mean connectivity was optimized. Using the dynamic tree cut algorithm, seven distinct gene coexpression modules were identified, with a TOM heatmap illustrating the network structure (Figures 2a, 2b, 2c, and 2d). The relationships between MEs and clinical traits were subsequently analyzed, and the green module exhibited the strongest correlation with endometriosis, encompassing 6869 genes (Supporting Information 1: Table S1). A detailed list of the genes in the green module is provided in Supporting Information 1: Table S1. Additionally, a strong positive correlation was observed between the MEs and genes within the green module (Figure 2e).

Figure 2WGCNA analysis of module–trait relationships and immune cell infiltration. (a) Heatmap showing the correlation between module eigengenes and clinical traits. Each row represents a different module (colored according to module designation), and the values represent the correlation (*r*) between the module and the trait. (b) Gene dendrogram and module colors obtained from hierarchical clustering of genes based on their expression profiles. The dynamic tree cut algorithm identified distinct modules that represent gene coexpression patterns. (c) Scale independence and mean connectivity analysis for the soft threshold power selection in the WGCNA analysis. (d) Heatmap of the topological overlap matrix (TOM) showing the network of coexpression between genes in the identified modules. Gene clusters with high similarity are grouped together. (e) Scatter plot showing the relationship between module membership in the green module and gene significance for endometriosis. (f) Boxplots of the immune cell fraction in normal and endometriosis samples. ns > 0.05;  ^∗^ *p* < 0.05;  ^∗∗^ *p* < 0.01;  ^∗∗∗^ *p* < 0.001.

**Figure:**
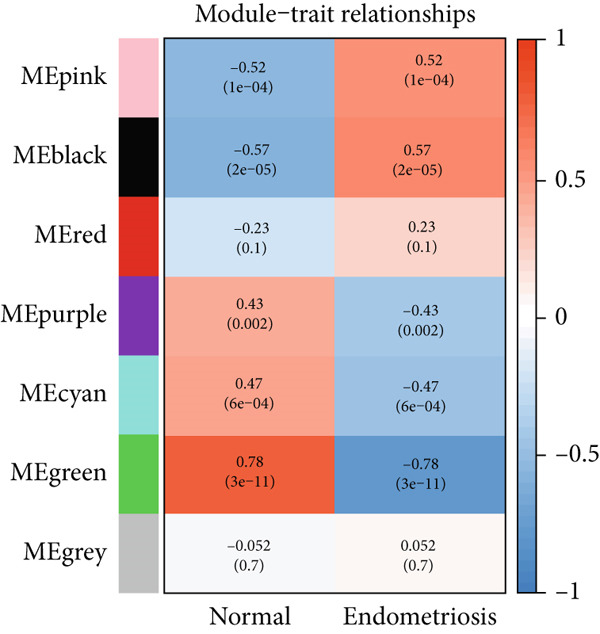
(a)

**Figure:**
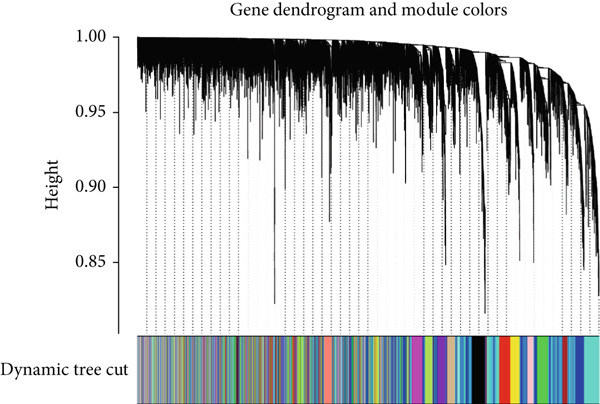
(b)

**Figure:**
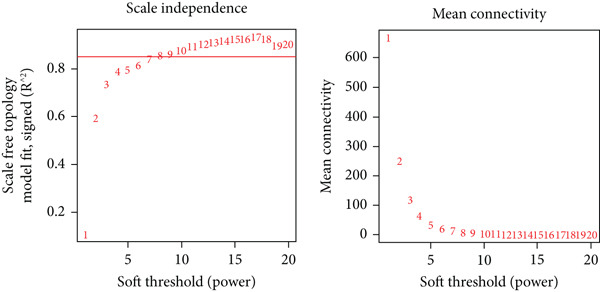
(c)

**Figure:**
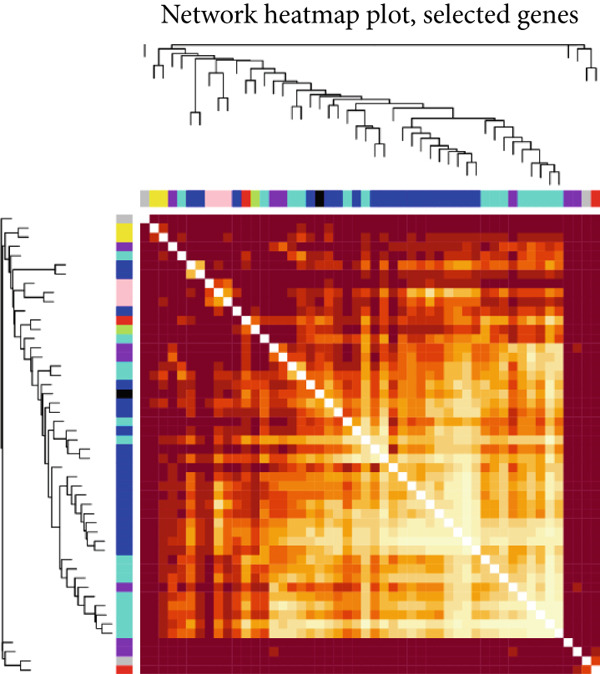
(d)

**Figure:**
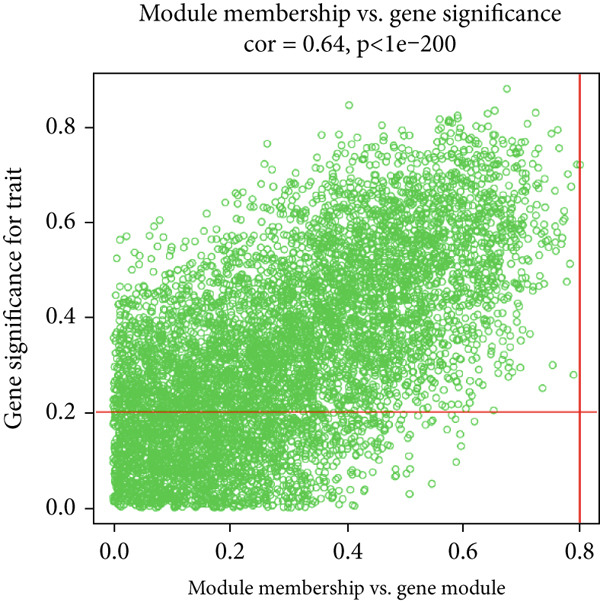
(e)

**Figure:**
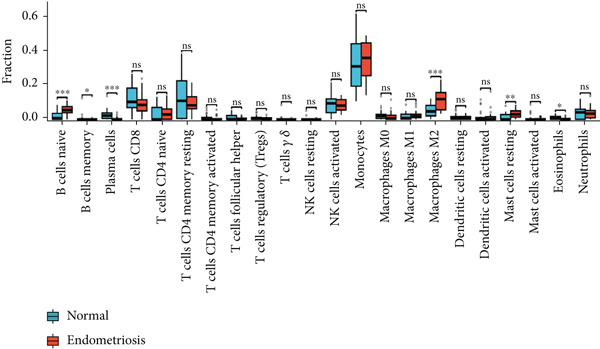
(f)

### 3.3. Immune Infiltration Analysis of Green Module Genes

We performed immune infiltration analysis of genes within the green module using the CIBERSORT algorithm to compare immune cell infiltration between normal samples and endometriosis samples (Figure 2f). The results indicated that overall differences in immune cell infiltration between the two groups were not significant. However, distinct differences were observed in the infiltration levels of naïve B cells, memory B cells, plasma cells, M2 macrophages, and eosinophils. In contrast, the infiltration levels of other immune cell types showed no significant variation between the two tissue types.

### 3.4. Acquisition and Enrichment Analysis of Hypoxia‐Related DEGs

By intersecting the DEGs from the merged datasets with the hypoxia‐related genes obtained from the GeneCards database, we identified 605 overlapping genes. Among these, 449 genes were both hypoxia‐related and differentially expressed, corresponding to the green module of interest (Figure 3a).

Figure 3Gene set enrichment and immune cell analysis in hypoxia‐related modules. (a) Venn diagram showing the overlap between DEGs related to hypoxia and the genes in the identified modules. (b) GO enrichment analysis of the hypoxia‐related DEGs. (c) GSEA of selected pathways. The plots show enrichment scores along with the corresponding *p* values and false discovery rates for each pathway. (d) Boxplots of immune cell fractions in normal and endometriosis samples across various immune cell types. ns > 0.05;  ^∗^ *p* < 0.05;  ^∗∗^ *p* < 0.01;  ^∗∗∗^ *p* < 0.001.

**Figure:**
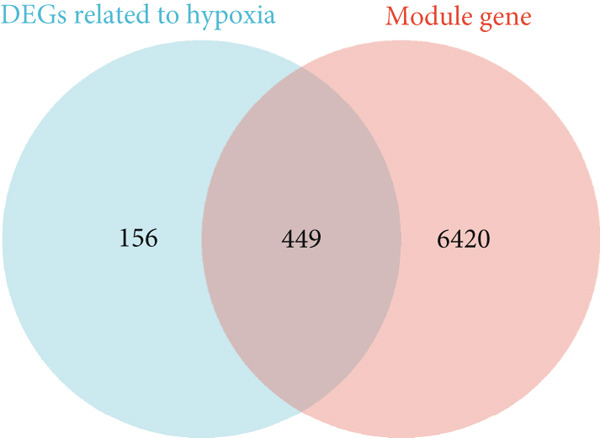
(a)

**Figure:**
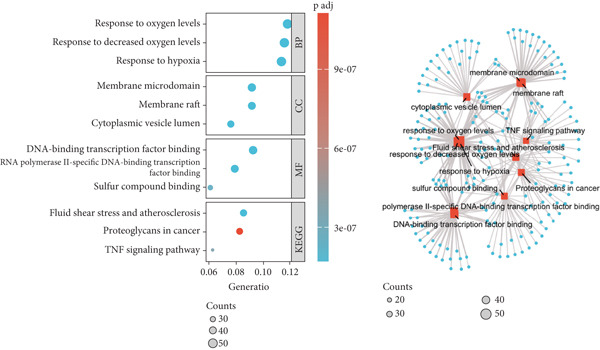
(b)

**Figure:**
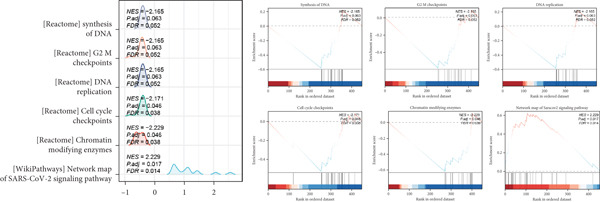
(c)

**Figure:**
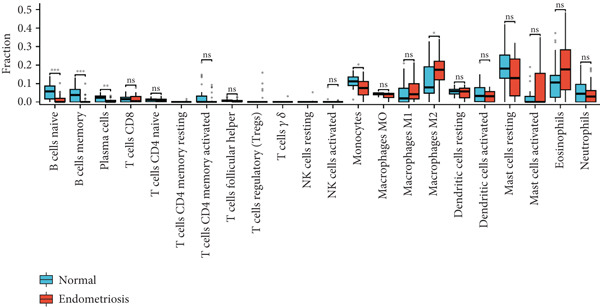
(d)

The results of GO and KEGG analyses of the 449 hypoxia‐related DEGs are shown in Figure 2b. Among the BP, CC, MF, and KEGG pathways, the three most enriched pathways were identified. They were response to decreased oxygen levels, response to hypoxia, response to oxygen levels, membrane raft, membrane microdomain, cytoplasmic vesicle lumen, RNA Polymerase II‐specific DNA‐binding transcription factor, DNA‐binding transcription factor, sulfur compound binding, fluid shear stress and atherosclerosis, TNF signaling pathway, and proteoglycans in cancer (Figure 3b) (Supporting Information 2: Table S2).

### 3.5. Hypoxia‐Related DEG Gene Set Enrichment Analysis (GSEA)

GSEA was conducted to identify the key biological pathways associated with endometriosis by comparing gene expression profiles. The enrichment results revealed several significantly dysregulated pathways, as shown in the GSEA ridge plot. Among the upregulated pathways, one displayed a normalized enrichment score (NES) of 2.229, with an adjusted *p* value (*p*.adj) of 0.017 and an FDR of 0.014, indicating a strong positive enrichment. Conversely, several pathways were significantly downregulated, with NES values ranging from −2.165 to −2.229 and corresponding *p*.adj values between 0.046 and 0.063. These pathways included critical BPs such as chromatin‐modifying enzymes, cell cycle checkpoints, DNA replication, and G2/M checkpoints, which are fundamental to cellular function and proliferation (Figure 3c) (Supporting Information 3: Table S3). We performed immune infiltration analysis of 449 genes using the CIBERSORT algorithm to compare immune cell infiltration between normal samples and endometriosis samples (Figure 3d). The results indicated that overall differences in immune cell infiltration between the two groups were not significant. However, distinct differences were observed in the infiltration levels of naïve B cells, memory B cells, plasma cells, monocytes, and M2 macrophages. In contrast, the infiltration levels of other immune cell types showed no significant variation between the two tissue types.

### 3.6. Machine Learning–Based Gene Selection

Based on our analysis, a total of 449 hypoxia‐related DEGs were identified. To identify genes with higher predictive value, we constructed machine learning models, including SVM, RF, GLM, and XGB, using the expression profiles of these genes. Among the models, SVM demonstrated the lowest root mean square of residuals (Figure 4a,b). The Top 10 most important variables for each model were ranked based on the root mean square error (RMSE) loss after permutation testing (Figure 4c). Based on these results, the SVM model, which showed the strongest discriminative performance, was chosen for further investigation.

Figure 4Feature selection and model evaluation for predicting endometriosis. (a) Cumulative distribution of the revenue for the four modes, with each feature′s contribution to the overall prediction model. (b) Boxplots of the selected features based on their importance across different models, showing the median, interquartile range, and outliers for each model. (c) Feature importance ranking obtained from several machine learning models, including GLM, RF, SVM, and XGBoost. (d) LASSO regression results showing the coefficient paths for various features as the fraction of the explained variance increases. (e) Coefficient plot from LASSO regression highlighting the regularization path and feature selection process. (f) The error rate was plotted against the number of selected features in the model, with the feature selection threshold identified at the point where the error rate was minimized. (g) Venn diagram showing the overlap between the features selected by the LASSO method and the SVM‐RFE method.

**Figure:**
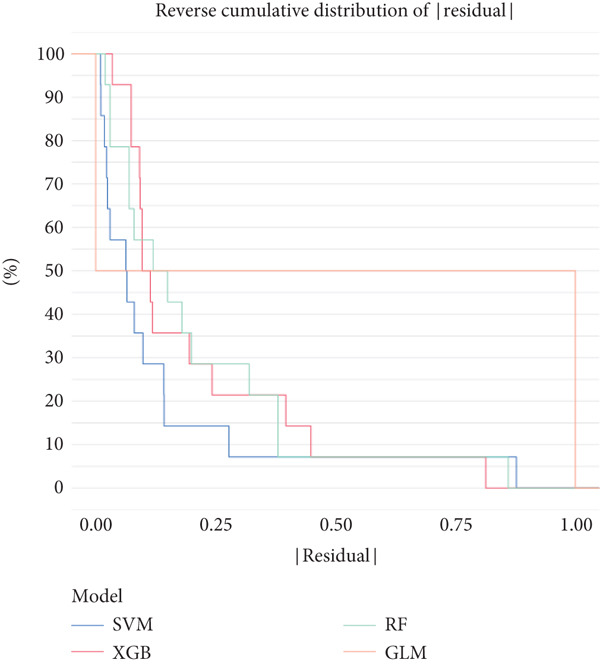
(a)

**Figure:**
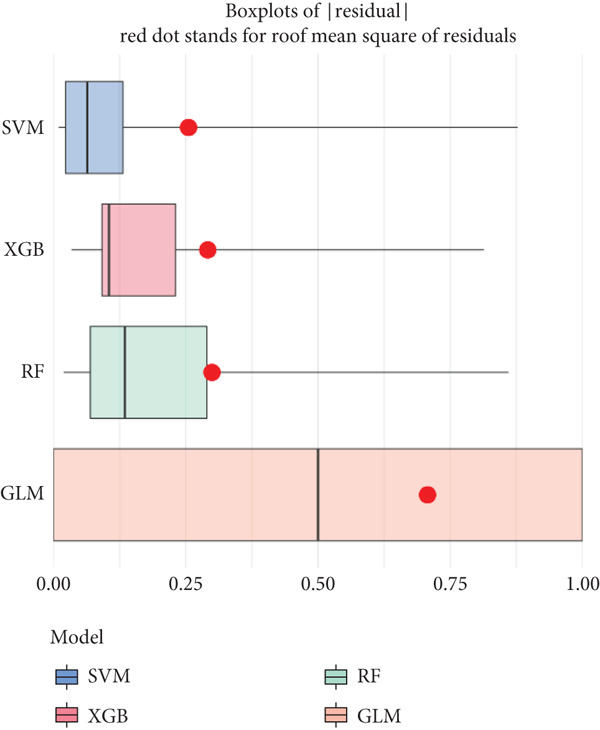
(b)

**Figure:**
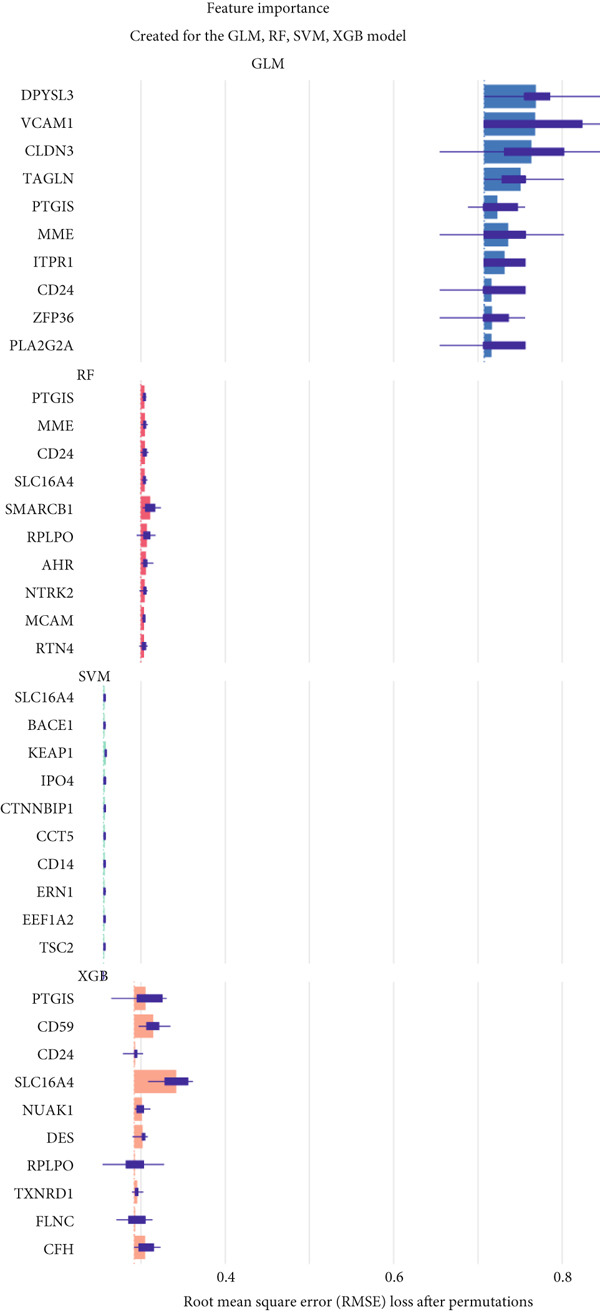
(c)

**Figure:**
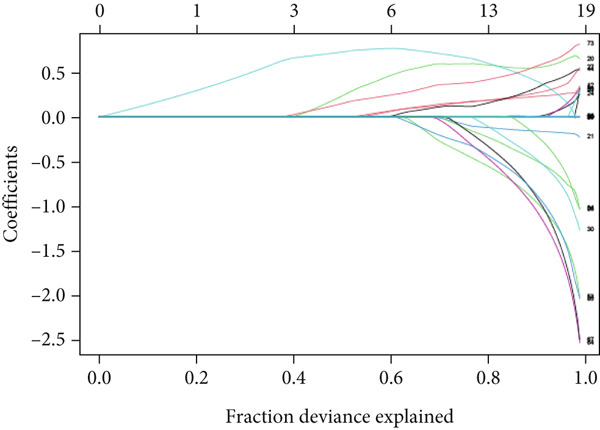
(d)

**Figure:**
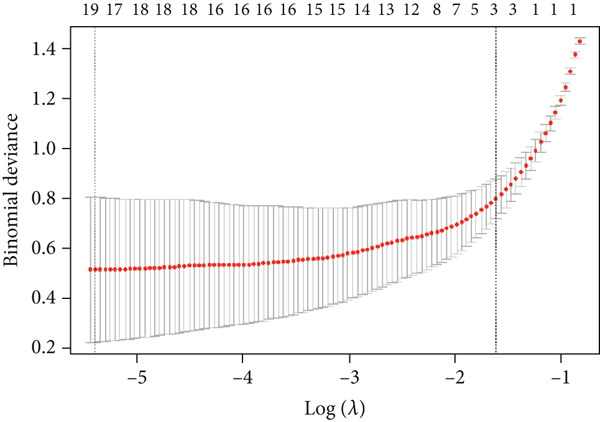
(e)

**Figure:**
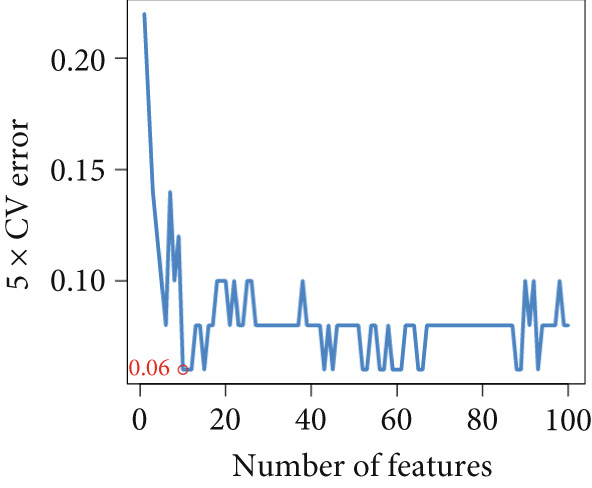
(f)

**Figure:**
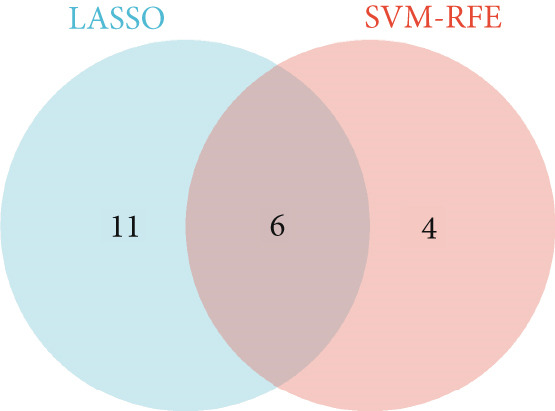
(g)

### 3.7. Selection of Hub Genes Using LASSO and SVM‐RFE

Following the identification of the optimal SVM model with the best discriminative performance based on the RMSE and permutation importance analysis, the Top 100 genes with the highest importance were selected from the SVM model for further investigation. These genes′ expression profiles were used to perform an integrated machine learning approach combining LASSO regression and SVM‐RFE to identify hub genes with the strongest predictive power.

LASSO regression was applied to narrow down the gene set by penalizing irrelevant features and retaining only those with nonzero coefficients, ensuring a robust and minimalistic gene panel. Subsequently, SVM‐RFE was used to rank the selected genes based on their contribution to the classification performance (Figures 4d, 4e, and 4f). Through this two‐step feature selection process, six hub genes were ultimately identified as the most critical predictors of endometriosis (Figure 4g). They were DDR2, Enolase 3 (ENO3), endothelial cell–specific Molecule 1 (ESM1), neuromedin B receptor (NMBR), protein kinase AMP‐activated noncatalytic subunit Beta 1 (PRKAB1), and pre‐mRNA processing Factor 19 (PRPF19). Figure 5a shows the chromosomal localization of these six genes. These hub genes exhibited high predictive significance, as determined by their consistent selection in cross‐validation and their contribution to the classification performance of the SVM model.

Figure 5Gene expression, correlation analysis, and predictive model evaluation. (a) Chromosomal localization of six genes. (b) Chord diagram illustrating the pairwise correlations between the genes. The correlation matrix shows values for all selected genes. (c) Forest plot showing the contribution of individual variables, including DDR2, ENO3, ESM1, NMBR, PRKAB1, PRPF19, total points, and linear predictors to the risk score model. (d) Boxplots of six gene expression levels in the merged dataset and validation dataset (GSE141519). (e) ROC curves for evaluating the predictive performance of the risk model based on the selected genes. The left plot shows the AUC values for the merged dataset, and the right plot shows the AUC values for the GSE141519 dataset. ns > 0.05;  ^∗∗^ *p* < 0.01;  ^∗∗∗^ *p* < 0.001.

**Figure:**
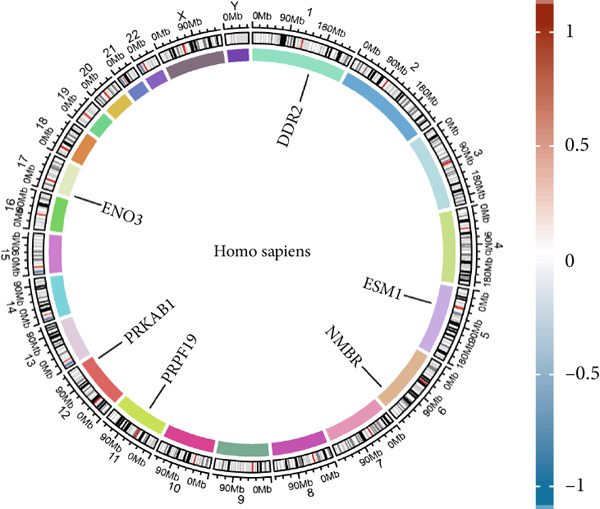
(a)

**Figure:**
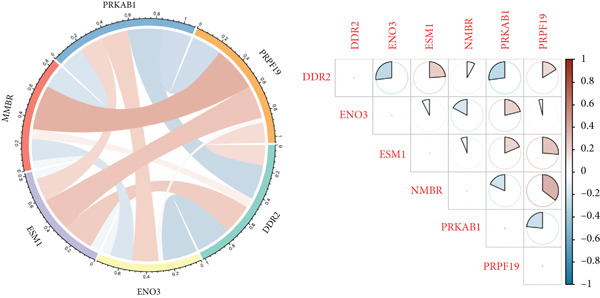
(b)

**Figure:**
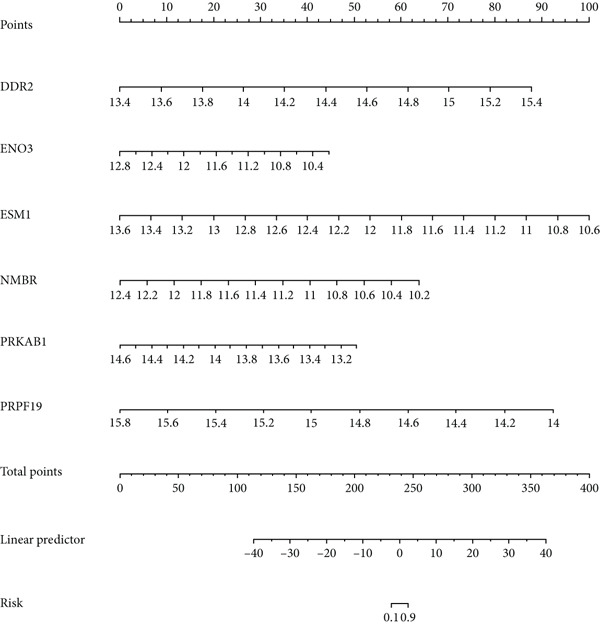
(c)

**Figure:**
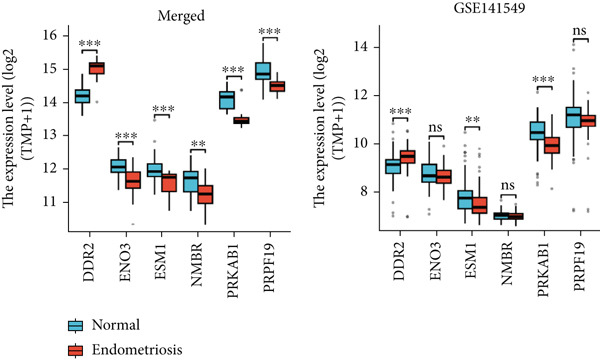
(d)

**Figure:**
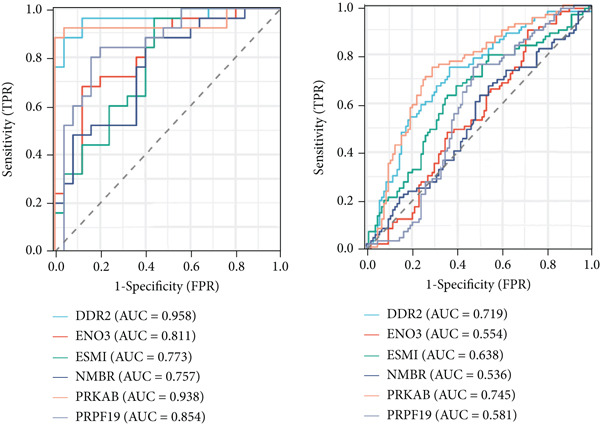
(e)

The gene expression analysis, visualized through heatmaps and circlize plots, revealed significant correlations between several key genes (Figure 5b). Notably, ENO3 and ESM1 exhibited a strong positive correlation, suggesting that they may be coregulated or involved in similar BPs. In contrast, DDR2 showed a moderate negative correlation with PRKAB1, indicating a potential inverse relationship in their expression patterns. Other gene pairs, such as NMBR and PRPF19, demonstrated weaker correlations, implying that these genes may function more independently or contribute to distinct regulatory pathways. The six hub genes, DDR2, ENO3, ESM1, NMBR, PRKAB1, and PRPF19, from the LASSO and SVM‐RFE model were enrolled as predictors for constructing a nomogram model (Figure 5c). Based on this model, we can infer the risk of endometriosis from the “total points.”

### 3.8. Validation of Hub Genes Using the GSE141549 Dataset

To validate the expression and diagnostic performance of the six identified hub genes, the GSE141549 dataset was used as an independent validation cohort. In the merged dataset, the expression levels of these six genes—DDR2, ENO3, ESM1, NMBR, PRKAB1, and PRPF19—showed significant differences between normal and endometriosis samples (DDR2, *p* < 0.001; ENO3, *p* < 0.001; ESM1, *p* < 0.001; NMBR, *p* = 0.002; PRKAB1, *p* < 0.001; PRPF19, *p* < 0.001). The diagnostic performance, assessed by ROC curve analysis, demonstrated high AUC values for the six genes: DDR2 (0.958), ENO3 (0.811), ESM1 (0.773), NMBR (0.757), PRKAB1 (0.938), and PRPF19 (0.854) (Figure 5d).

In the GSE141549 validation cohort, only DDR2, ESM1, and PRKAB1 maintained the same expression trends as observed in the merged dataset, with statistically significant differences between normal and endometriosis samples (DDR2, *p* < 0.001; ESM1, *p* = 0.002; PRKAB1, *p* < 0.001). The other three genes (ENO3, NMBR, and PRPF19) did not exhibit significant differential expression between the two groups in the validation dataset (ENO3, *p* = 0.212; NMBR, *p* = 0.409; PRPF19, *p* = 0.062).

The diagnostic performance of these genes in the validation set showed variability. The ROC AUC values were as follows: DDR2 (0.719), ENO3 (0.554), ESM1 (0.638), NMBR (0.536), PRKAB1 (0.745), and PRPF19 (0.581). Among these, DDR2 exhibited consistent diagnostic potential across both the merged and validation datasets, while PRKAB1 also showed relatively stable performance. Based on its robust diagnostic efficacy and significant differential expression, DDR2 was selected for further study as the key hub gene (Figure 5e).

### 3.9. Impact of DDR2 Knockdown on Proliferation, Invasion, and Migration in Endometriosis Cells

To assess the impact of DDR2 knockdown on the proliferative capacity of endometriosis cells VK2/E6E7 and 12Z, we conducted CCK‐8 and EdU assays. In VK2/E6E7 cells, the OD450 values in the DDR2 knockdown group were consistently lower than those in the control group. Specifically, at 24, 48, and 72 h, the OD450 values for the DDR2 knockdown group (siDDR2: 24 h, OD450 = 1.046 ± 0.009; 48 h, OD450 = 1.559 ± 0.017; 72 h, OD450 = 1.584 ± 0.025) were significantly reduced compared to the control group (siNC: 24 h, OD450 = 1.086 ± 0.010; 48 h, OD450 = 1.609 ± 0.010; 72 h, OD450 = 1.713 ± 0.023) (24 h, *t* = 3.005, *p* = 0.009; 48 h, *t* = 2.584, *p* = 0.022; 72 h, *t* = 3.784, *p* = 0.002) (Figure 6a).

Figure 6Impact of DDR2 knockdown on proliferation, invasion, and migration in endometriosis cells. (a) The CCK‐8 assay demonstrates that DDR2 knockdown resulted in consistently lower OD450 values in both VK2/E6E7 and 12Z cells, compared to the control group. (b) The EdU incorporation assay reveals that the EdU‐positive cell rate in VK2/E6E7 and 12Z cells was significantly decreased following DDR2 knockdown, compared to the control group. (c) The Transwell invasion assay shows that the invasive capacity of VK2/E6E7 and 12Z cells was significantly reduced in the DDR2 knockdown group compared to the control group. (d) The Transwell migration assay demonstrates that the migratory ability of VK2/E6E7 and 12Z cells was significantly diminished following DDR2 knockdown, as compared to the control group. ns > 0.05;  ^∗^ *p* < 0.05;  ^∗∗^ *p* < 0.01;  ^∗∗∗^ *p* < 0.001.

**Figure:**
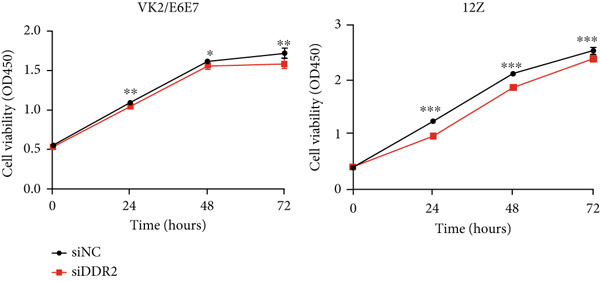
(a)

**Figure:**
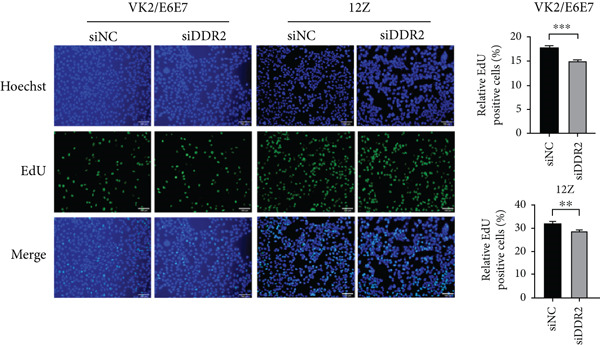
(b)

**Figure:**
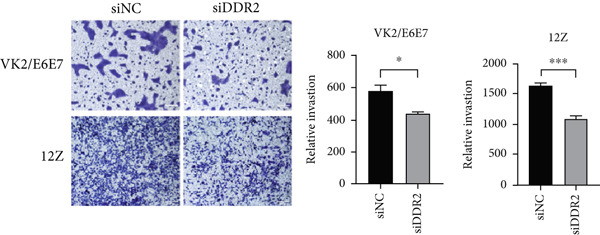
(c)

**Figure:**
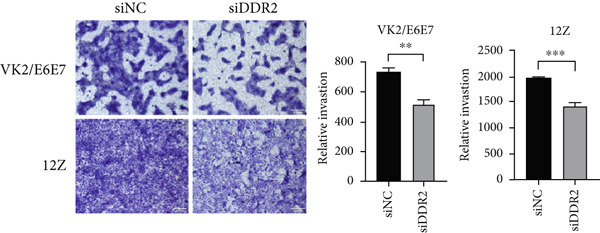
(d)

Similarly, in 12Z cells, the DDR2 knockdown group exhibited significantly lower OD450 values than the control group at all time points. At 24, 48, and 72 h, the OD450 values in the DDR2 knockdown group (siDDR2: 24 h, OD450 = 0.977 ± 0.004; 48 h, OD450 = 1.865 ± 0.014; 72 h, OD450 = 2.389 ± 0.019) were substantially reduced compared to the control group (siNC: 24 h, OD450 = 1.244 ± 0.011; 48 h, OD450 = 2.115 ± 0.009; 72 h, OD450 = 2.530 ± 0.019), with statistically significant differences observed at each time point (24 h, *t* = 22.700, *p* < 0.001; 48 h, *t* = 15.280, *p* < 0.001; 72 h, *t* = 5.167, *p* < 0.001) (Figure 6a).

Subsequent EdU assays were conducted to further investigate cell proliferation. The results demonstrated that, following DDR2 knockdown, the EdU‐positive cell rate in VK2/E6E7 cells (15.011 ± 0.222) was significantly lower than that observed in the control group (17.848 ± 0.317) (*t* = 7.328, *p* < 0.001). Similarly, in 12Z cells, the EdU‐positive cell rate in the DDR2 knockdown group (28.952 ± 0.565) was significantly reduced compared to the control group (32.401 ± 0.847) (*t* = 3.386, *p* = 0.003) (Figure 6b).

To assess the impact of DDR2 on the invasive potential of endometriosis cells, Transwell invasion assays were conducted. The results revealed that in VK2/E6E7 cells, the invasion capacity of the DDR2 knockdown group (435.00 ± 17.210) was significantly lower compared to the control group (577.75 ± 38.915) (*t* = 3.355, *p* = 0.015). Similarly, in 12Z cells, the invasive ability of the DDR2 knockdown group (1103.5 ± 31.077) was markedly reduced compared to the control group (1652.5 ± 17.400) (*t* = 15.41, *p* < 0.001) (Figure 6c).

To further examine the role of DDR2 in cell migration, Transwell migration assays were performed. The results indicated that in VK2/E6E7 cells, the migratory ability of the DDR2 knockdown group (514.00 ± 30.100) was significantly impaired compared to the control group (730.50 ± 23.782) (*t* = 5.644, *p* = 0.001). Likewise, in 12Z cells, the migratory capacity of the DDR2 knockdown group (1439.0 ± 34.044) was substantially reduced relative to the control group (1981.3 ± 6.170) (*t* = 15.67, *p* < 0.001) (Figure 6d).

### 3.10. Interactions Between DDR2 and Drugs

*Arum ternatum* Thunb., a commonly used therapeutic agent for endometriosis, contains baicalein, cavidine, sitogluside, and stigmasterol as its active components. Molecular docking simulations were conducted using AutoDock Vina to assess the interactions between DDR2 and the four core active compounds. The most optimal protein–ligand docking complexes were selected, resulting in nine protein–ligand structures. In all docking simulations, the best binding energy (affinity) values were less than −7 kcal/mol, indicating strong binding affinities for DDR2 with each active compound (Supporting Information 3: Table S3).

Near the binding pocket of DDR2, the amino acids Y703, A653, L616, and A774 of DDR2 can form hydrophobic interactions with baicalein. The amino acids T701 and D775 of DDR2 can form hydrogen bonds with baicalein, while F776 of DDR2 can participate in *π*‐stacking and hydrophobic interactions with baicalein (Figure 7a). Near the DDR2′s binding pocket, the amino acids L616, V624, Y703, and L764 of DDR2 can form hydrophobic interactions with cavidine (Figure 7b). Additionally, near DDR2′s binding pocket, the amino acids L764, A774, D775, F776, I685, K655, L616, A653, and Y703 can form hydrophobic interactions with sitogluside. The amino acids E705, Y703, and N706 of DDR2 near the binding pocket can form hydrogen bonds with sitogluside (Figure 7c). Finally, near DDR2′s binding pocket, the amino acids Y703, A653, L764, F776, V624, and L616 can form hydrophobic interactions with stigmasterol (Figure 7d). These results quantify the strength of the interactions and reinforce the potential of DDR2 as a target for therapeutic intervention.

Figure 7Molecular interactions and electrostatic surface potentials of protein–ligand complexes. (a–d) Molecular interactions between DDR2 and different ligands: (a) baicalein, (b) cavidine, (c) sitogluside, and (d) stigmasterol. Each panel includes 2D representations of protein–ligand interactions (left), 3D structures of the complexes (middle), and electrostatic surface potentials of the complexes (right), with key amino acids and bond formations highlighted.

**Figure:**
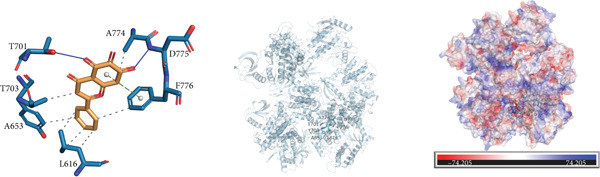
(a)

**Figure:**
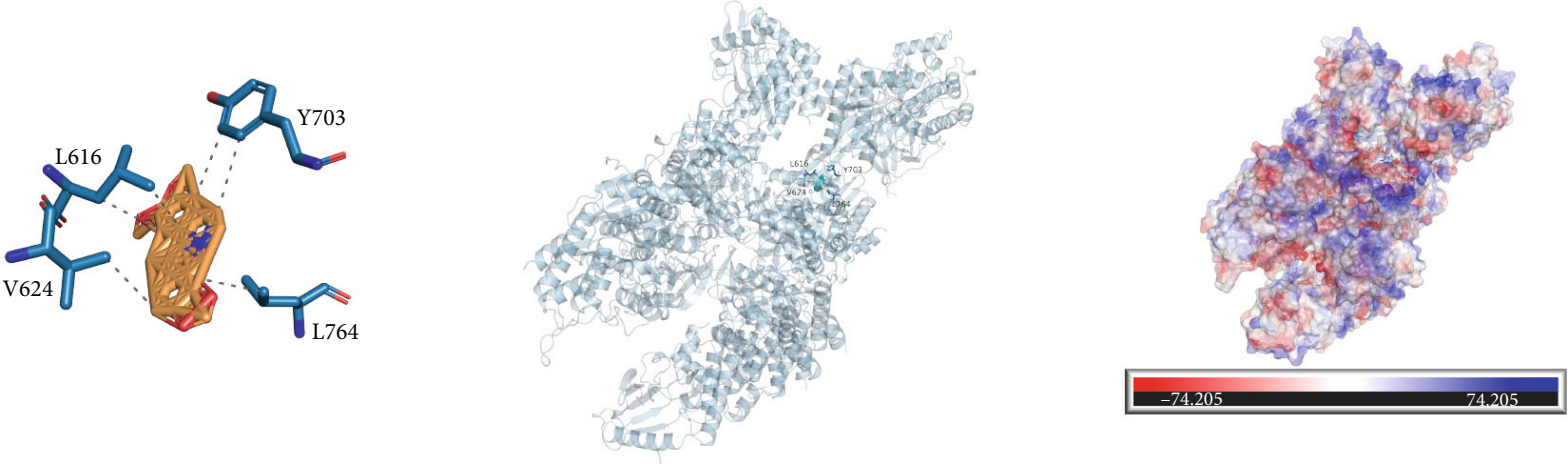
(b)

**Figure:**
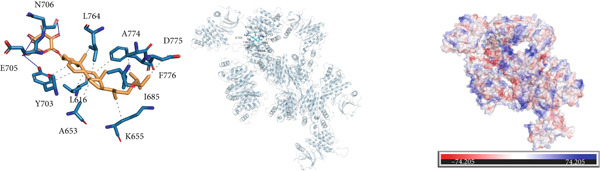
(c)

**Figure:**
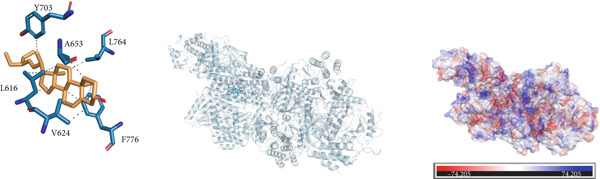
(d)

## 4. Discussion

This study comprehensively explored the role of hypoxia‐related genes in endometriosis through the integration of GEO datasets and hypoxia‐associated genes from the GeneCards database, identifying 449 DEGs enriched in hypoxia‐related biological pathways. A multistep selection process pinpointed six hub genes (DDR2, ENO3, ESM1, NMBR, PRKAB1, and PRPF19), with DDR2 emerging as a key candidate due to its consistent expression profile, robust diagnostic performance, and functional relevance. In the merged dataset, DDR2 showed a marked differential expression between normal and endometriosis tissues. Functional experiments revealed that DDR2 knockdown significantly suppressed endometriosis cell proliferation, migration, and invasion, highlighting its pivotal role in hypoxia‐driven pathophysiological processes. These findings establish DDR2 as a promising biomarker and therapeutic target for endometriosis.

Elevated expression of hypoxia‐related genes has been consistently observed in ectopic versus eutopic endometrial tissues, underscoring the central role of hypoxia in endometriosis pathophysiology. Key markers such as HIF‐1*α* and VEGF are hallmarks of ectopic lesions, driving angiogenesis and tissue remodeling [27]. Hypoxia‐responsive microRNAs, including miR‐210, are also significantly upregulated, facilitating cell survival and autophagy under hypoxic conditions [23]. Furthermore, genes involved in tissue remodeling, such as TWIST and MMPs, exhibit elevated expression in ectopic lesions, emphasizing the invasive and proliferative potential driven by the hypoxic microenvironment [22]. Hypoxia‐related biomarkers hold diagnostic and prognostic promise; HIF‐1*α* overexpression correlates with disease severity, while systemic markers such as VEGF, IL‐6, and oxidative stress indicators like 8‐OHdG provide minimally invasive options for disease detection [28]. HIFs, particularly HIF‐1*α*, orchestrate these responses by stabilizing under hypoxia and activating downstream pathways critical for lesion persistence and expansion [29]. HIF‐1*α* upregulates VEGF to promote angiogenesis and enhances MMP (MMP‐2 and MMP‐9) expression, facilitating ECM degradation and lesion migration [30]. Estrogen further amplifies HIF‐1*α* activity via the PI3K/Akt pathway, creating a synergistic effect between hypoxia and hormonal signaling [31]. Dysregulated angiogenesis perpetuates localized hypoxia, creating a self‐sustaining cycle, while progesterone resistance exacerbates HIF‐1*α*‐driven inflammation and remodeling [31–33].

DDR2, a collagen‐binding receptor tyrosine kinase, is critically involved in regulating cellular adhesion, migration, and invasion, particularly through its role in ECM remodeling. DDR2′s interaction with fibrillar collagen triggers downstream signaling pathways, prominently activating MMPs, including MMP‐2 and MMP‐9, which facilitate ECM degradation and promote invasive behavior [34].

This study highlights DDR2 as a crucial player in the hypoxic microenvironment of endometriosis, with significant implications for both diagnosis and therapy. DDR2, a collagen‐binding receptor tyrosine kinase, regulates ECM remodeling and cellular invasiveness under hypoxic conditions. Mechanistically, DDR2 binds fibrillar collagen, triggering downstream signaling pathways critical for regulating cellular adhesion, migration, and invasion. Within the hypoxic microenvironment characteristic of endometriosis, DDR2 likely acts in synergy with HIF‐1*α*, enhancing processes such as tissue remodeling and angiogenesis. Previous studies have demonstrated DDR2′s role in upregulating MMPs, particularly MMP‐2 and MMP‐9, which degrade the ECM and facilitate the invasive potential of ectopic lesions into surrounding tissues, consistent with its observed role in promoting invasiveness in endometriotic cells [35, 36].

Notably, DDR2 interacts with key signaling pathways that overlap with well‐established therapeutic targets in endometriosis, such as VEGF and HIF‐1*α*. VEGF, a potent angiogenic factor, is critical for the formation of new blood vessels in ectopic endometrial lesions, a process vital for their growth and survival [37, 38]. Hypoxia‐induced stabilization of HIF‐1*α* enhances VEGF expression, thereby promoting angiogenesis and tissue remodeling [33]. Given DDR2′s role in modulating the ECM and promoting invasiveness, it is likely to act synergistically with VEGF and HIF‐1*α* in driving lesion progression in endometriosis. Moreover, DDR2′s relationship with HIF‐1*α* further emphasizes its potential as a therapeutic target. As a hypoxia‐responsive gene, DDR2 is regulated by HIF‐1*α*, which not only enhances DDR2 expression but also amplifies its downstream effects on ECM remodeling and cell motility [39, 40]. This interplay between DDR2 and HIF‐1*α* underscores the importance of targeting DDR2 in conjunction with other hypoxia‐related pathways to manage disease progression in endometriosis. Furthermore, DDR2′s involvement in the inflammatory environment of endometriotic lesions—mediated through its interaction with proinflammatory cytokines such as IL‐6 and TNF‐*α*—adds another layer of complexity to its therapeutic potential [41–44]. Hypoxia‐driven activation of DDR2 amplifies inflammatory responses, sustaining the chronic inflammatory microenvironment of ectopic lesions. By targeting DDR2, it may be possible to not only inhibit ECM remodeling and cell migration but also modulate the inflammatory responses that drive chronic pain and infertility in endometriosis patients.

Taken together, DDR2′s dual role in regulating tissue remodeling and inflammation, combined with its interactions with established therapeutic targets like VEGF and HIF‐1*α*, positions DDR2 as a promising novel target for therapeutic intervention in endometriosis. Collectively, these findings highlight DDR2′s multifaceted role in supporting the persistence, invasiveness, and progression of endometriosis, making it a key driver of the disease′s pathological features [45].

The results from both the CCK‐8 and EdU assays demonstrated a significant reduction in the proliferative capacity of VK2/E6E7 and 12Z cells following DDR2 knockdown. The OD450 values, which reflect cell viability and metabolic activity, were consistently lower in the DDR2 knockdown groups at all time points in both cell lines. This reduction suggests that DDR2 is involved in regulating cell proliferation. In VK2/E6E7 cells, the EdU‐positive cell rate was significantly lower in the DDR2 knockdown group, indicating that DDR2 knockdown affects DNA synthesis and cell cycle progression. Similarly, in 12Z cells, the EdU‐positive cell rate was also reduced following DDR2 knockdown, further supporting the role of DDR2 in regulating cell proliferation. These findings are consistent with previous studies that have linked DDR2 to cellular processes such as cell cycle regulation and proliferation. Additionally, the Transwell invasion and migration assays revealed that DDR2 knockdown significantly impaired both the invasive and migratory abilities of VK2/E6E7 and 12Z cells. In both assays, the DDR2 knockdown groups demonstrated substantially reduced cell invasion and migration compared to the control groups, suggesting that DDR2 may be involved in the regulation of cell motility and the ability of endometriosis cells to invade surrounding tissues. This is particularly relevant in the context of endometriosis, where abnormal cell invasion and migration are key features of the disease′s pathology. In summary, these findings suggest that DDR2 may be a potential therapeutic target for modulating the growth and spread of endometriotic lesions.

This study has certain limitations. Future investigations should aim to explore the molecular mechanisms underlying DDR2 in greater detail. Furthermore, animal models and clinical trials are necessary to validate DDR2′s involvement in endometriosis and to evaluate its potential as a therapeutic target.

## 5. Conclusion

This study identified DDR2 as a key hypoxia‐related gene in endometriosis, demonstrating its diagnostic and therapeutic potential through integrated analyses and experimental validation. These findings provide a foundation for future research and clinical applications aimed at improving endometriosis diagnosis and treatment.

## Ethics Statement

This study did not involve human participants, animal subjects, or any personal data that would require ethics approval. Therefore, ethics approval and consent to participate were not applicable.

## Disclosure

All authors have read and approved the final manuscript and consent to its publication.

## Conflicts of Interest

The authors declare no conflicts of interest.

## Author Contributions

Mingqi Zhao and Xiaoqin Lu were responsible for designing the study. Mingqi Zhao, Panpan Zhao, and Xiaoqin Lu wrote the manuscript, extracted and analyzed data, interpreted the results, and conducted the experiments. Caiyi Wang, Dan Ren, and Yuxia Song contributed to editing the article. Caiyi Wang and Xiaoqin Lu contributed to reviewing the article. Mingqi Zhao and Panpan Zhao contributed equally to this work and share first authorship.

## Funding

No funding was received for this manuscript.

## Supporting Information

Additional supporting information can be found online in the Supporting Information section.

## Supporting information


**Supporting Information 1** Table S1: Genes in green module.

**Supporting Information 2** Table S2: The results of GO and KEGG.

**Supporting Information 3** Table S3: The results of GSEA and the results of molecular docking.

## Data Availability

The data that support the findings of this study are available in the supporting information of this article.
